# Raised BMI cut-off for overweight in Greenland Inuit – a review

**DOI:** 10.3402/ijch.v72i0.21086

**Published:** 2013-08-05

**Authors:** Stig Andersen, Karsten Fleischer Rex, Paneeraq Noahsen, Hans Christian Florian Sørensen, Gert Mulvad, Peter Laurberg

**Affiliations:** 1 Arctic Health Research Centre, Aalborg University Hospital, Aalborg, Denmark; 2 Department of Medicine, Queen Ingrids Hospital, Nuuk, Greenland; 3 Department of Geriatric Medicine, Aalborg University Hospital, Aalborg, Denmark; 4 Ammassalik Hospital, Ammassalik, Greenland; 5 Primary Health Care Clinic, Nuuk, Greenland; 6 Department of Endocrinology, Aalborg University Hospital, Aalborg, Denmark

**Keywords:** BMI, cut-off point, overweight, obesity, Greenland Inuit, ethnicity, review

## Abstract

**Background:**

Obesity is associated with increased morbidity and premature death. Obesity rates have increased worldwide and the WHO recommends monitoring. A steep rise in body mass index (BMI), a measure of adiposity, was detected in Greenland from 1963 to 1998. Interestingly, the BMI starting point was in the overweight range. This is not conceivable in a disease-free, physically active, pre-western hunter population.

**Objective:**

This led us to reconsider the cut-off point for overweight among Inuit in Greenland.

**Design and findings:**

We found 3 different approaches to defining the cut-off point of high BMI in Inuit. First, the contribution to the height by the torso compared to the legs is relatively high. This causes relatively more kilograms per centimetre of height that increases the BMI by approximately 10% compared to Caucasian whites. Second, defining the cut-off by the upper 90-percentile of BMI from height and weight in healthy young Inuit surveyed in 1963 estimated the cut-off point to be around 10% higher compared to Caucasians. Third, if similar LDL-cholesterol and triglycerides are assumed for a certain BMI in Caucasians, the corresponding BMI in Inuit in both Greenland and Canada is around 10% higher. However, genetic admixture of Greenland Inuit and Caucasian Danes will influence this difference and hamper a clear distinction with time.

**Conclusion:**

Defining overweight according to the WHO cut-off of a BMI above 25 kg/m^2^ in Greenland Inuit may overestimate the number of individuals with elevated BMI.

The on-going epidemic of obesity is associated with complications such as psychosocial disease states, muscle and joint disorders, diabetes, increased cancer risk, cardiovascular disease, and all-cause mortality (1–5). Thus, obesity is a major threat that may slow or even reverse the gains in life expectancy that have been achieved over the past decades.

It is important to identify people at risk both at the individual level in everyday clinical practice and at a population level to identify a population hazard and guide preventative measures.

The guidelines issued by the WHO have defined overweight and obesity as a body mass index (BMI) of 25 kg/m^2^ or higher and 30 kg/m^2^ or higher, respectively (2). These cut-off points are derived from morbidity and mortality data from predominantly Caucasian populations in Europe and the US (2,6). However, ethnic differences in body build may exist (7) that may render cut-off points inappropriate (8). Thus, the mean population level of BMI is lower in Asian than in European and US populations while the prevalence of type-2 diabetes is high (8,9). Also, a high level of mean population BMI has been found in pre-western Greenland Inuit (10) that has been associated with a low occurrence of type-2 diabetes (11). Such data on ethnic differences have added to the questioning of whether the WHO defined cut-off points can be generalised to non-Caucasian populations (6,12–14).

## Body build and BMI in Inuit

Inuit body build differs from that of Caucasians in that Inuit have larger torsos and shorter limbs (15,16). This increases BMI independent of the degree of body fat as the torso carries more weight per centimetre than legs (17), and 31% of young, fit hunters in Greenland investigated around 1963 were classified as overweight when using the WHO cut-off points (10). Two different approaches have been used to quantify the impact on BMI of this difference in body build as discussed below.

### Sitting-height/height ratio

Shorter legs relative to the torso increase the ratio of sitting height to standing height. This is calculated as the sitting height divided by total height, also known as the Cormic index (17). Inuit have a higher length of torso relative to the legs and hence a higher sitting-height/height ratio compared to non-Inuit whites (16,18,19). This influences BMI towards higher values (17,20). Consequently, BMI may overestimate the prevalence of overweight and obesity in Inuit populations compared to other populations. This may be corrected for by identifying and using the BMI cut-off values in Inuit that detects the same degree of adiposity and risk profile as the BMI cut-off of 25 and 30 kg/m^2^ does in Caucasians.

Charbonneau-Roberts et al. reported a sitting height/height ratio of 0.54 for Inuit while it was 0.52 for non-Inuit (18). Norgan calculated the influence of sitting-height/height ratio on BMI from anthropometric measurements on 18,000 individuals (17). By using his estimate of an increase of 0.8 kg/m^2^ for men and 1.2 kg/m^2^ for women for each 0.01 increment in sitting-height/height ratio, the BMI that corresponds to 25 kg/m^2^ in non-Inuit whites is around 10% higher in Inuit (see Table I).

**Table I:** The BMI in Inuit that corresponds to a BMI of 25 kg/m^2^ in non-Inuit whites as estimated by different methods: Noahsen assessed Inuit BMI based on plasma lipids

Population	Measure	BMI	Reference	
Non-Inuit whites	Reference	25	WHO	(2)
Inuit men	HDL	27.62	Jørgensen, Noahsen	(22,31)
	Triglycerides	27.18	Jørgensen, Noahsen	(22,31)
	HDL	28.36	Young, Noahsen	(23,31)
	Triglycerides	26.36	Young, Noahsen	(23,31)
	Distribution	27.9	Andersen	(10)
	SH/H ratio	26.6	Chateau-Degat, Norgan	(15,17)
Inuit women	HDL	26.54	Jørgensen,Noahsen	(22,31)
	Triglycerides	27.18	Jørgensen,Noahsen	(22,31)
	HDL	27.93	Young, Noahsen	(23,31)
	Triglycerides	27.27	Young, Noahsen	(23,31)
	Distribution	27.7	Andersen	(10)
	SH/H ratio	27.4	Chateau-Degat, Norgan	(15,17)

Andersen assessed Inuit BMI based on BMI distributions. Norgan assessed BMI based on sitting height/height ratios (31). Noahsen computed cut-off points based on data extracted from comparative studies of BMI and plasma lipids (22,23).

### BMI distribution in pre-western Inuit

A way to define an optimal BMI for a population is to identify a healthy population that is not malnourished and estimate a BMI norm from this population. To do this among Greenland Inuit requires access to height, weight, physical examination, and data on the occurrence of disease in pre-western Inuit.

**Fig. 1:**
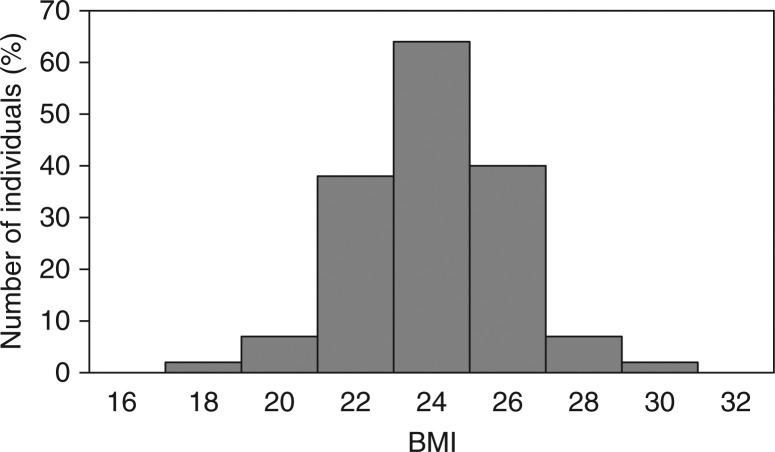
Distribution of BMI among 20–29-year-old men in East Greenland in 1963 (n=176). The WHO definition of overweight renders 31% of young hunters overweight. Defining overweight by an Inuit 90-percentile sets the cut-off point at 27.9 kg/m^2^ in Inuit men (from ref. 10).

A comprehensive population-based study of Greenland Inuit was performed in 1962–1964, prior to the westernisation of Greenlandic societies (11). The original data sheets from East Greenland were kept and donated to Queen Ingrids Hospital in Nuuk, Greenland, by the descendants of the late Jørgen Littauer. The data covered 96.9% of the population of East Greenland (n =1,852) and included height, weight, medical history and a physical examination (21). Andersen and colleagues calculated BMI for evaluation of overweight and obesity (10). They found that BMI in healthy 20–29 year old men displayed a symmetrical distribution that is unique for biological data (Fig. 1) (10). According to the WHO definition, 31% of the young hunters were rendered overweight. This is not likely considering that the Inuit hunters around 1963 lived a physically very active life that depended on hunting and fishing from kayaks in the Arctic sea where excess body fat would be a hazardous drawback. Also, excessive food intake was limited to short periods that were regularly followed by periods of food shortages. Still, malnourishment was prevented by the distribution of food through trading posts during famine. Also, systematic illnesses were prevented as public health care was established in East Greenland in the late 1940s.

Based on the symmetrical distribution, Andersen and colleagues calculated 90-percentile cut-off points for defining overweight in healthy Inuit men and women. These cut-off points were around 10% higher than those defined by the WHO (see Table I).

## LDL-cholesterol, triglycerides and BMI

The impact of BMI on plasma lipids differed between Inuit and non-Inuit in the 2 studies that included both Inuit and non-Inuit and thus allowed for a comparative analysis (22,23). This may be explained in 2 ways. The higher plasma lipids in Inuit could be a marker of environmental and genetic factors not related to overweight that contributed to a difference in the cardiovascular risk between Inuit and non-Inuit. This interpretation was supported by the lower occurrence of ischemic heart disease among pre-western Greenland Inuit compared to Caucasian populations (24,25). However, the occurrence of ischemic heart disease among Greenland Inuit has risen markedly (25,26) in parallel with the transition of societies in Greenland towards a more sedentary lifestyle and a change in dietary habits with a higher intake of imported foods (27,28). This towards a more This challenges the former explanation.

A different interpretation is that it is not the lipids but rather the BMI that differs between Inuit and non-Inuit. This is in keeping with the high level of physical activity in pre-western Inuit that contributed to the low occurrence of ischemic heart disease in pre-western Inuit (29) and the subsequent rise in ischemic heart disease as the sedentary lifestyle gains ground (30).

Based on the assumption that Inuit and non-Inuit tend to have similar levels of lipids and that the differences observed relate to overweight, it is possible to calculate the Inuit BMI that gives the same lipid profile as a BMI of 25 kg/m^2^ in non-Inuit. This was carried out by Noahsen (31). Data reported from Greenland by Jørgensen et al. (22) and from Canada by Young (23) were used to calculate regression equations for the association between BMI and HDL-cholesterol and triglycerides in Inuit and in non-Inuit (31). The Inuit BMI corresponding to the non-Inuit BMI of 25 kg/m^2^ was calculated for both HDL-cholesterol and triglycerides in men and women. Values are given in Table I. A clear pattern was seen with a BMI of around 10% higher in Inuit compared to non-Inuit for the same degree of dyslipidaemia. These levels are in accordance with the levels suggested from estimations of body build (Table I).

## Discussion

Three different approaches were used to evaluate the influence of Inuit ethnicity on BMI cut-off points. Independently they suggested that BMI cut-offs for overweight and obesity should be 10% higher in Inuit compared to non-Inuit whites. These uniform findings all point towards a higher BMI in Inuit for the same degree of risk of disease that is in keeping with the finding that the degree of metabolic disturbances is lower at the same BMI in Inuit compared with Caucasians (22,23,32).

Only one anthropometric measure of adiposity was considered in this analysis. The prediction of disease risk can be done from a number of measures of excess body fat. Some are direct and some are indirect measures.

Computer tomography and dual energy X-ray absorptiometry are direct measures of body fat but both are cumbersome and associated with exposure to radiation that increases the risk of cancer, mainly in the former as dual energy X-ray absorptiometry only causes limited radiation exposure. Still, these methods are not appropriate for everyday clinical practice or large-scale clinical studies. Bioelectrical impedance is another approach that has been used in a single study of Canadian Inuit (15). It requires equipment and is based on the assumption that the body is a cylindrical-shaped ionic conductor with non-adipose tissue as resistor and capacitor, which is influenced by tissue hydration (33) and ethnicity (34). Hence, caution should be taken when evaluating results and the method should be validated in Inuit. Also, these direct measures of the amount of body fat are not able to assess the metabolic effect of the fat detected. There are, for example, ethnic differences in the compartments of abdominal adipose tissue, visceral and subcutaneous abdominal adipose tissue (35) that may differ in metabolic effects (36). The distribution of these compartments did not differ between Inuit and non-Inuit whites (35) but may differ between superficial and deep subcutaneous adipose tissue (36). In addition, Inuit host metabolically active brown adipose tissue (37) that has a different metabolic effect compared to white adipose tissue (38). Still, there is an association between the amount of excess body fat and the risk of ischemic heart disease, as well as an association between excess body fat and anthropometric measures of adiposity (39,40).

Anthropometric measures are simple and easy to obtain. They include BMI and waist–hip-ratio that assess total body adiposity, while waist circumference describes central adiposity. They are all indirect measures of adiposity and have an inherited imprecision in identifying metabolic risk. Still, they have a predictive value for metabolic syndrome and vascular health (3,5,39,40).

We chose to focus on BMI as a model for analysing the influence of ethnicity on anthropologic measures of adiposity. There is some agreement between the different anthropometric measures (41) but the data should not be extended to cover other measures of body fat. Nevertheless, they draw attention to the importance of ethnicity in the assessment of overweight and obesity among Inuit.

## Conclusion

A BMI cut-off point of 25 kg/m^2^ that defines overweight in non-Inuit corresponded to a BMI cut-off point of approximately 27.5 kg/m^2^ in Inuit as estimated using 3 independent approaches. The relatively higher BMI cut-off point among Inuit compared to non-Inuit and the lower impact of high BMI on metabolic indicators suggest the need for a higher BMI cut-off in Inuit for the same degree of disease risk.

However, the association is complex. It is modified by the genetic admixture of Inuit and non-Inuit that over time may cause an underestimation of obesity rates if rigorous BMI cut-off points are used for all populations in Greenland. It may be useful to include the degree of Inuit heritage in the evaluation of metabolic risk when using BMI.

None of the 3 approaches reviewed here can be used to settle ethno-specific BMI cut-off points and the association with different rates of diseases related to obesity remains to be determined in prospective studies with diseases as outcome variables.
